# Prediction of the Dental Arch Perimeter in a Kurdish Sample in Sulaimani City Based on Other Linear Dental Arch Measurements as a Malocclusion Preventive Measure

**DOI:** 10.1155/2020/8869996

**Published:** 2020-12-21

**Authors:** Fadil Abdullah Kareem, Aras Maruf Rauf, Arass Jalal Noori, Trefa M. Ali Mahmood

**Affiliations:** Department of Pedodontics, Orthodontics and Preventive Dentistry, College of Dentistry, University of Sulaimani, P.O. Box 334, Sulaymaniyah 46001, Iraq

## Abstract

The current study aimed to find a prediction equation to estimate the arch perimeter (AP) depending on various arch dimensions including intercanine width (ICW), intermolar width (IMW), interpremolar width (IPMW), and arch length (AL) in a sample of the Kurdish population in Sulaimani City. The study sample was 100 pairs of preorthodontic dental casts. Calculations of dental arch dimensions and perimeter were performed by a digital vernier. Statistical analysis was performed via using the SPSS version 25 software. The developed prediction equation for the upper arch was Y = +1.3 × (arch length) + 1 × (intermolar width), whereas the equation for the lower arch was Y = +0.9 × (intermolar width) + 0.92 × (intercanine width). Paired *t*-test revealed no statistical difference between predicted and real arch perimeters. Two separate prediction equations for upper and lower arches were developed based on the arch length (AL) and intermolar width (IMW) for the maxillary arch, intermolar (IMW), and inter canine widths (ICW) for the lower arch. The developed equations could have further beneficial impacts on orthodontic diagnosis and treatment planning.

## 1. Introduction

Dental arch perimeter is regarded as one of the most vital dental arch parameters for orthodontic diagnosis and treatment planning. It is defined as the distance from the mesial surface of the first permanent molar around the dental arch to the same point in the opposite side [1].

Various methods for measuring the dental arch perimeter have been adopted by several authors: these calculation methods include direct measurement by extending a brass or steel wire along the distances to be measured and then measuring the length of the wire after straightening it [2] using a segmented arch technique to be calculated on a study cast [3], usage of a special device named a catenometer to measure dental arch perimeter [4], the use of sonic dental cast digitization in calculating mesiodistal teeth width and perimeter [5], and finally, computing the arch perimeter by mathematical method via different equations and functions [6–8].

Dental arch and soft tissue parameters can be considered age-dependent factors in untreated persons with orthodontic appliances [9]. The greatest increase in the upper arch perimeter occurs during the mixed dentition period which may be ascribed to the permanent incisor eruption as well as to the largest mesiodistal width of primary molars compared to premolars [10], with a decreased perimeter being observed with advanced age [11]. Accordingly, many authors [12, 13] have reported an increase in dental arch perimeter until permanent dentition completion that is followed by a diminution of this dimension with age, especially in the mandibular arch [14].

A smaller arch perimeter of deciduous dentition in contemporary samples was observed than in historic samples of children 50 years ago as reported by Warren and Bishara in 2001 [15]. Ricketts et al. reported in 1982 [16] that for each one-millimetre increase of intercanine and intermolar width, the arch length increased by 1 mm and 0.25 mm, respectively. Therefore, this significant relationship between arch width and perimeter can be exploited in favour of prediction of expecting dental arch perimeters by the use of various arch dimensions.

Until now, several prediction equations have been developed [17, 18] to estimate dental arch perimeter from different arch dimensions including IMW and ICWs. An equation was developed by Sanin et al. in 1970 [6] as dental arch perimeter = (dental arch width × 0.504) + (dental arch length × 1.525) + 14.856. Another equation was proposed by Paulino et al. in 2008 [7], based on a Spanish population and utilizing intercanine width: arch length (perimeter) = (1.36 × intercanine width) + 29.39. In 2014, Al-Khatieeb et al. [8] made an attempt to find a prediction equation of arch perimeter depending on arch width at the level of each tooth for both the maxillary and the mandibular arch. Finally, Al-Ansari et al., in 2019 [19], investigated other equations on a sample of Iraqi Arab population for both the maxillary arch: 23.597 + (ICW × 1.040) + (AL × 0.378), and the mandibular arch: 23.644 + (ICW × 0.645) + (IMW × 0.356) + (AL × 0.221).

The expectation of arch perimeter from arch widths and length must be considered in the space analysis, individual, and general features of management of malocclusion. Additionally, proper treatment planning, especially in cases of dental arch expansion, and stability of treatment end result are regarded as other crucial considerations. Moreover, no study has been conducted on the Kurdish population in respect of this crucial issue. Thus, the purpose of the current study was to find a prediction equation to estimate the AP depending on various arch dimensions including ICW, IMW, IPMW, and PD in a Kurdish sample in Sulaimani City. There was a null hypothesis of no association of different arch dimensions with dental arch perimeter.

## 2. Materials and Methods

### 2.1. Study Population

This cross-sectional study was approved by the ethical committee of medical colleges/University of Sulaimani (ethical number = 400) in accordance with the Helsinki declaration. The study sample was composed of 100 pairs of dental casts of untreated orthodontic patients attending private orthodontic clinics in Sulaimani City on a retrospective cross-sectional study basis. The ages of participants were in the range of 14-24 years old. The criteria of sample selection included the following: Class I angle relationship of less than 3 mm crowding and spacing; complete permanent set from first permanent molar of one side to the same teeth of the other without gingival enlargement; no previous orthodontic, orthopaedic, or orthognathic treatment; no dental anomalies; no obvious proximal loss of teeth; and no open bite and crossbite.

### 2.2. Measurements

**Figure 1:**
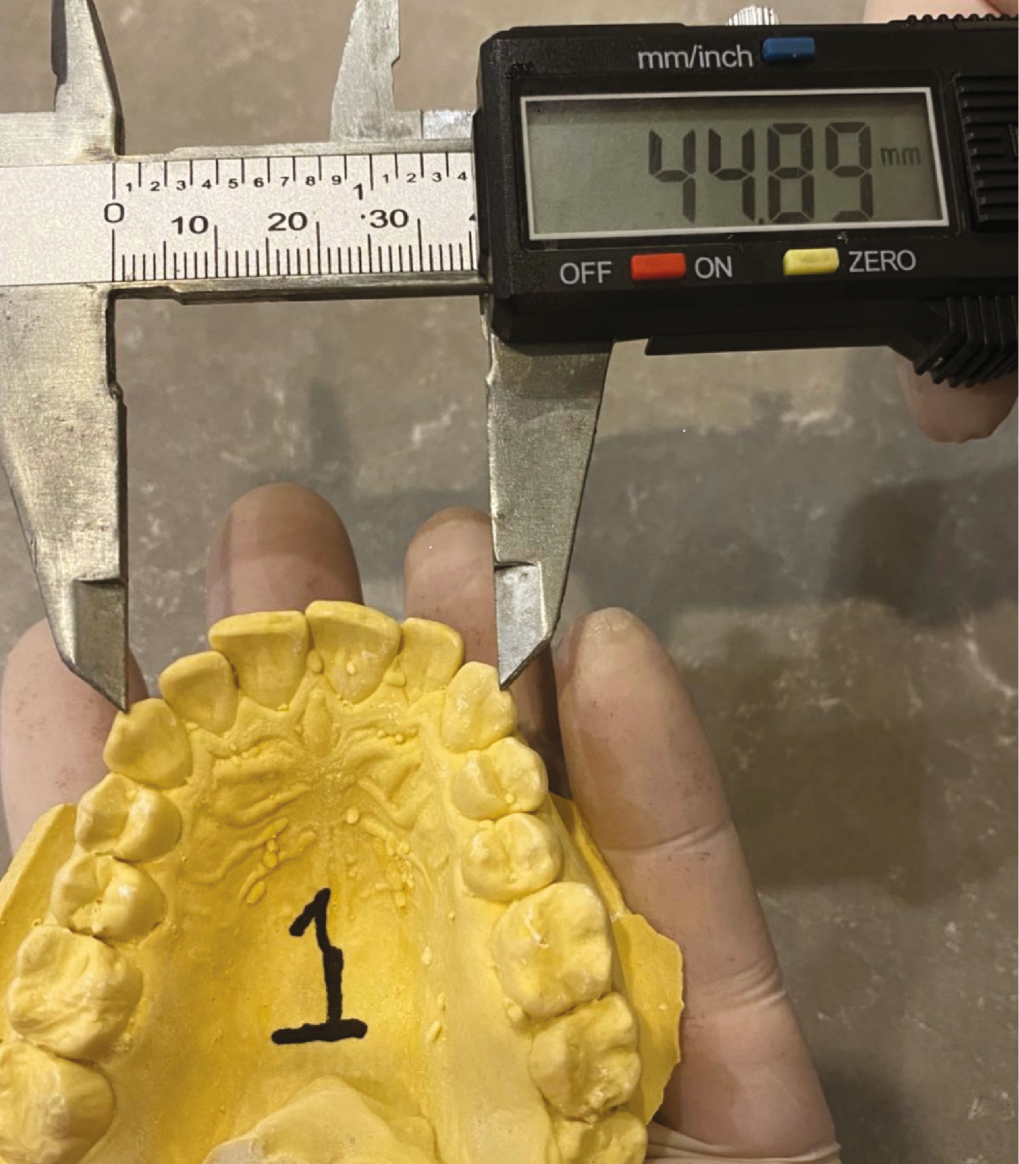
Digital vernier.

Linear measurements: the linear measurements were calculated via a digital vernier with an accuracy of 0.01 mm (Mitutoyo, Japan) (Figure 1) which included the following:

1. Intermolar width: the linear distance at the level of the molar mesiobuccal cusp tips [20]
2. Interpremolar width: the linear distance at the level of the premolar buccal cusp tips [21]
3. Intercanine width: the linear distance at the level of the canine cusp tips [21]
4. Arch length: the vertical distance between the central points of the right and left central incisors and the line connecting the mesiobuccal cusp tips of the first molars [22]
5. Arch perimeter: the dental arch perimeter was obtained by addition of measurements of five segments: from the mesial point of the first molars to the distal point of the canines, from the distal point of the canines to the distal point of the central incisors on the right and left sides, and from the distal point of the right central incisors to the distal point of the left central incisors [16]

### 2.3. Statistical Analysis

The continuous data was subjected to Shapiro-Wilk normality test prior to data analysis. Inter- and intraexaminer calibration was carried out on 10 randomly selected study casts. Paired *t*-test was adopted to test for the differences in both inter- and intraexaminer calibrations. The collected data were subjected to descriptive and inferential statistical analysis through the use of the Statistical Package for the Social Sciences (SPSS) software version 25.

Pearson's test of correlation coefficient was used to determine the relationship of the dental arch perimeter with each dental arch width and length. Stepwise regression analysis was used to find the predictor(s) of dental arch perimeters. After the application of regression equations, the actual and predicted arch perimeters were compared using a paired sample *t*-test. The probability value (*P* value) was set at 0.05.

## 3. Results

The descriptive analysis of the whole study sample was explained in Table 1. The results of upper arch statistical tests revealed a strong positive correlation of arch length (*r* = 0.769) with AP, followed by a moderately strong positive correlation of each of the intermolar widths (*r* = 0.670) and intercanine (*r* = 0.640) widths, in one hand. On the other hand, a weak positive correlation was found concerning each of the first and second IPMWs. Therefore, the suggested upper arch perimeter equation prediction of the present study was highly reliant on upper AL and IMW. Regarding the lower dental arch, the findings revealed a strong correlation of intermolar width (*r* = 0.708) and moderately strong correlation of intercanine width (0.684). Hence, the suggested lower arch perimeter equation prediction of the present study was highly reliant on IMW and ICW (Table 2).

The stepwise regression analysis of the AL and IMW (the independent variables) for the upper arch and IMW and ICWs for the lower arch (the independent variables) which were used to predict the dental arch perimeter (the dependent variable) are represented by the following equations (Table 3):

(1) Upper arch:
  (1) $$\begin{array}{c} Y=+B1X1+B2X2,\\ Y=+1.3\text{x}\left(X1\right)+1\text{x}\left(X2\right), \end{array}$$

*Y*: the value of the dependent variable which is the arch perimeter

*B*1, *B*2: the regression coefficients of each variable, respectively

*X*1, *X*2: the values of independent variables (predictors) which are the different arch widths (dental arch length and intermolar widths, respectively)

(2) Lower arch:
  (2) $$\begin{array}{c} Y=+B1X1+B2X2,\\ Y=+0.9\text{x}\left(X1\right)+0.92\text{x}\left(X2\right), \end{array}$$

*Y*: the value of the dependent variable which is the arch perimeter

*B*1, *B*2: the regression coefficients of each variable, respectively

*X*1, *X*2: the values of independent variables (predictors) which are the different arch widths (intermolar width and intercanine widths, respectively)

Afterwards, a paired *t*-test was used in comparison between the real and predicted arch perimeters (Table 4) which indicated no significant difference between the two at (*P* > 0.05) level.

## 4. Discussion

The present study was an attempt to find a correlation between APs with different linear arch dimensions in patients who had not been subjected to orthodontic treatment as this type of treatment may affect the original measurements [23]. Hence, the findings cannot be compared to those of studies conducted on patients who had previously undergone orthodontic treatment. Additionally, knowledge of orthodontic professionals concerning the dental arch changes that happen during growing years and after in normal untreated persons is crucially providing baseline data from which to plan treatment therapy [9]. Also, Defraia et al. in 2006 stated that different ethnic groups and populations display variable dental arch measurements and characteristics [24]. As a result, possession of a reliable prediction method of the arch perimeter is highly advisable for each ethnic group. Thus, the current study was designed and conducted.

An increase of arch perimeter was observed at a mixed dentition period until completion of permanent dentition then become diminished with age particularly in the lower arch [14]. And that is why two separate arch perimeter prediction equations were proposed by the current study: one for the upper arch and the other for the lower arch.

Study casts were selected as a raw material for conduction of the study as it can offer much information about the intended case, and it has also many advantages, such as determination of Bolton's ratios, space available and required calculation, arch widths, lengths, and perimeters, with the aid of vernier or OrthoCad as analyzing software [25].

The cross interaction between upper and lower dental arches in a manner whereby they act together implies the constitution of a single biologic unit, indicating that changes in each are directly related to those in the other. Consequently, a strong correlation between various linear dimensions within the same dental arch is expected. Additionally, the findings of the present study indicated a strong correlation of AL and IMW in the upper arch and strong correlation of both IMW and ICWs in the lower arch with the AP. The existence of a strong correlation between two variables estimates the size of one of the possible by knowing the dimensions of the other. The obtained predicted dental arch perimeter values were compared with the actual ones using a paired sample *t*-test, and the results revealed no significant difference.

Low correlations of upper first and second premolars with the upper arch perimeters (*r* = 0.413) and (*r* = 0.438) were found, respectively. Also, correlations of lower first and second premolars with lower arch perimeter (*r* = 0.575) and (*r* = 0.442) were observed, respectively, indicating no linear correlation. Accordingly, they were undependable for the development of the expected equation for the arch perimeter in both arches.

In a study by Paulino et al. in 2008 [7], the highest correlation was observed between arch PM and ICW in both arches, implying that changes in one magnitude may be directly related to changes in the other, which was in agreement with the present study findings, while a weak correlation was found between upper and lower AP and upper and lower IMW, respectively, which was in disagreement with the results of the present study. Another study by Tibana et al. in 2004, a weak correlation between ICW and AL and the strong correlation between upper ICW and lower AL were observed [26].

The current study is considered to be the first study conducted on a Kurdish sample and only the fifth study around the world to predict arch perimeter from arch lengths and widths. Some authors developed one equation for both arches, whereas separate equations were developed for the upper and lower arches by the present study. Further studies on cases of Class I malocclusion with more than 3 mm crowding or spacing and Class II and Class III malocclusions are recommended to be undertaken in the future.

## 5. Limitations

To overcome any possible limitation of the present study, a longitudinal study is highly advisable to follow up the consecutive developmental changes, acquiring the real dimensions then comparing the predicted measurements with the real value.

## 6. Conclusions

Utilization of upper arch length, upper and lower intermolar width, and lower inter canine width to establish new regression equations to predict upper and lower arch perimeters was the most crucial achievement of the current study. Furthermore, no significant differences were identified between predicted and actual perimeters. The findings of this study should be considered during space analysis, malocclusion treatment, and in particular in proper treatment during arch expansion. Accordingly, the developed equations could have further beneficial impacts on diagnosis and treatment planning.

## Figures and Tables

**Table 1:** Descriptive statistics for the whole study sample.

Maxillary arch					
	*N*	Minimum	Maximum	Mean	Std. deviation
Intermolar width	100	44.36	56.33	50.42	2.92
Interfirst premolar width	100	39.42	50.47	45.96	2.67
Intersecond premolar width	100	38.25	45.53	41.58	2.25
Intercanine width	100	29.44	37.36	33.85	2.12
Arch length	100	14.48	24.47	18.39	2.13
Arch perimeter	100	64.71	81.73	74.35	3.49

Mandibular arch					
Intermolar width	100	40.25	50.37	44.58	2.94
Interfirst premolar width	100	34.22	46.22	39.52	3.17
Intersecond premolar width	100	30.00	39.20	34.33	2.34
Intercanine width	100	24.00	30.51	26.43	2.02
Arch length	100	10.31	17.40	14.02	2.16
Arch perimeter	100	58.04	71.10	64.63	3.64

**Table 2:** Correlation between perimeter and other linear arch dimensions.

Arch perimeter	Intermolar width	1^st^ interpremolar	2^nd^ interpremolar	Intercanine width	Arch length
Maxillary	0.670	0.4123	0.438	0.640	0.769
Mandibular	0.708	0.575	0.442	0.684	0.273

**Table 3:** Regression equations for dental arch prediction.

Arch	Regression equations	*r*	*R* ^2^	*P* value
Maxillary arch	Arch length × 1.3 + intermolar width	0.813	0.661	0.001
Mandibular arch	Intermolar width × 0.9 + intercanine width × 0.92	0.746	0.556	0.001

**Table 4:** Comparison between actual and predicted arch perimeters.

Arch	Value	Descriptive statistics		*t*-test	*P* value
		Mean	Sd		
Maxillary	Actual	74.35	3.499	0.052	0.958
	Predicted	74.33	5.02		

Mandibular	Actual	64.63	3.642	0.675	0.501
	Predicted	64.44	4.22		

## Data Availability

The data used to support the findings of this study are obtained from private orthodontic clinics in Sulaimani City/Kurdistan/Iraq and are available from the corresponding author upon request.
